# Naloxone interventions in opioid overdoses: a systematic review protocol

**DOI:** 10.1186/s13643-019-1048-y

**Published:** 2019-06-11

**Authors:** Lindsay Victoria Shaw, Jessica Moe, Roy Purssell, Jane A. Buxton, Jesse Godwin, Mary M. Doyle-Waters, Penelope M. A. Brasher, Jeffrey P. Hau, Jason Curran, Corinne M. Hohl

**Affiliations:** 1 School of Social Dimensions of Health, University of Victoria, 3800 Finnerty Road, Victoria, BC V8P 5C2 Canada; 2 Canadian Institute for Substance Use Research, 2300 McKenzie Ave, Victoria, BC V8P 5C2 Canada; 3 Department of Emergency Medicine, University of British Columbia, 855 West 12th Avenue, Vancouver, BC V5Z 1M9 Canada; 4 Vancouver General Hospital, 855 West 12th Avenue, Vancouver, BC V5Z 1M9 Canada; 5 BC Centre for Disease Control, 655 West 12th Avenue, Vancouver, BC V5Z 4R4 Canada; 6 School of Population and Public Health, 2329 West Mall, Vancouver, BC V6T 1Z4 Canada; 7 Centre for Clinical Epidemiology and Evaluation, Vancouver Coastal Health Research Institute, 828 West 10th Avenue, Vancouver, BC V5Z 1M9 Canada

**Keywords:** Naloxone, Drug overdose, Adverse effects, Drug administration schedule, Drug dosage calculations, Administration and dosage, Fentanyl, Systematic review, Protocol

## Abstract

**Background:**

North America is in the midst of an unabated opioid overdose epidemic due to the increasing non-medical use of fentanyl and ultra-potent opioids. Naloxone is an effective antidote to opioid toxicity, yet its optimal dosing in the context of fentanyl and ultra-potent opioid overdoses remains unknown. This review aims to determine the relationship between the first empiric dose of naloxone and reversal of toxicity, adverse events, and the total cumulative dose required among patients with undifferentiated opioid overdoses and those with suspected toxicity from ultra-potent opioids. Secondary objectives include evaluating the relationship between the cumulative naloxone dose and toxicity reversal and adverse events, among patients with undifferentiated opioid overdoses and those with suspected toxicity from ultra-potent opioids.

**Methods:**

To identify studies, we will search MEDLINE, Embase, CENTRAL, DARE, CDAG, CINAHL, Science Citation Index, multiple trial registries, and the gray literature. Included studies will evaluate patients with suspected or confirmed opioid toxicity from undifferentiated opioids and ultra-potent opioids, who received an empiric and possibly additional doses of naloxone. The main outcomes of interest are the relationship between naloxone dose and toxicity reversal and adverse events. We will include controlled and non-controlled interventional studies, observational studies, case reports/series, and reports from poison control centers. We will extract data and assess study quality in duplicate with discrepancies resolved by consensus or a third party. We will use the Downs and Black and Cochrane risk of bias tools for observational and randomized controlled studies. If we find sufficient variation in dose, we will fit a random effects one-stage model to estimate a dose-response relationship. We will conduct multiple subgroup analyses, including by type of opioid used and by suspected high and low prevalence of ultra-potent opioid use based on geographic location and time of the original studies.

**Discussion:**

Our review will include the most up-to-date available data including ultra-potent opioids to inform the current response to the opioid epidemic, addressing the limitations of recent reviews. We anticipate limitations relating to study heterogeneity. We will disseminate study results widely to update overdose treatment guidelines and naloxone dosing in Take Home Naloxone programs.

**Electronic supplementary material:**

The online version of this article (10.1186/s13643-019-1048-y) contains supplementary material, which is available to authorized users.

## Background

Opioids are a powerful class of drugs that inhibit the transmission of pain signals to the brain and spinal cord [1, 2]. As non-opioid pharmaceutical options to relieve pain are limited, clinicians often prescribe opioids to manage painful medical and surgical conditions [3, 4]. However, opioids also produce feelings of euphoria, contributing to their abuse potential and can lead to tolerance resulting in the need for escalating doses and physical dependence [5, 6]. Once dependent, people who use opioids may misuse prescription drugs or acquire drugs on the illicit market. The rise in prescribed opioids over the past 20 years has been associated with increasing misuse and rising opioid-related deaths [7–13].

Fentanyl and other ultra-potent opioids have been found in a substantial proportion of recent opioid overdoses and are thought to be the driver of the current epidemic of overdose deaths [14–16]. They are between 50 and 10,000 times more potent than heroin [17–19]. When people who use drugs consume a more potent drug than they are habituated to, they commonly experience rapid respiratory and central nervous system depression and may die or survive with anoxic brain damage [2].In British Columbia (BC), the epicenter of the Canadian opioid epidemic, fentanyl was detected in 93% of the heroin supply in and was present in 81% of the 1156 fatal overdoses recorded in 2017 [20, 21].

Naloxone can reverse opioid toxicity if administered immediately after the onset of respiratory depression [22]. It is a competitive opioid antagonist and displaces opioids from receptors, reversing respiratory depression and coma. However, if administered in too high of a dose, or if repeat doses are administered too rapidly, naloxone may precipitate acute opioid withdrawal syndrome consisting of vomiting, tachycardia, shivering, sweating, and tremor. Additional serious adverse effects include pulmonary edema [23–32], hypertensive emergencies, ventricular dysrhythmias [33], delirium [34], seizures [34], and death [35–38]. Even if no immediate life-threatening adverse events occur, patients experiencing acute opioid withdrawal become agitated and commonly require sedation with other agents, putting them at risk for aspiration and recurrent respiratory depression [39, 40]. If patients in acute withdrawal are not sedated, they may leave the hospital against medical advice and use opioids again in an attempt to treat their withdrawal symptoms [41]. This puts them at risk for cumulative opioid toxicity as naloxone is a short-acting agent and usually wears off before the first opioid has been eliminated [42, 43]. Recently, naloxone-induced acute opioid withdrawal has become more common, with 9% of patients reporting severe and 18% reporting moderate withdrawal symptoms when administered in a community setting [44].

To effectively reverse opioid toxicity while avoiding acute opioid withdrawal and other adverse effects, the optimal dose of naloxone needs to be determined. However, no consensus exists on the optimal starting dose or route of administration: the American Heart Association recommends administering 2 mg of naloxone intranasally, or 0.4 mg intramuscularly, while other guidelines recommend an initial dose of 0.05 mg intramuscularly [45, 46]. Determining the optimal starting dose is complicated by the variation in type and dose of opioid causing toxicity [5, 47]: while the mean cumulative naloxone dose required to reverse heroin toxicity is 0.9 mg intravenously, the mean dose to reverse fentanyl toxicity has been reported as 3.4 mg and may range up to 12.0 mg [48–50]. In one randomized trial comparing 2 mg of intramuscular naloxone with the same intranasal dose, both routes of administration were equally effective, while other investigators observed that intranasal administration was associated with a delay in its onset of action and that it was not effective in reversing transdermal fentanyl toxicity [51–54].

While reviews on naloxone dosing have been completed, they excluded data from ultra-potent opioid toxicity [16, 55–63]. Therefore, the results of prior reviews have limited generalizability to treatment protocols in jurisdictions currently being inundated with ultra-potent opioids.

### Objectives

Our main objectives are to synthesize the available evidence on the relationship between the empiric first dose of naloxone administered and (1) the proportion of patients experiencing effective reversal of opioid toxicity, (2) the proportion of patients experiencing serious adverse events, and (3) the cumulative dose of naloxone administered in cases of both undifferentiated and suspected ultra-potent opioid toxicity. Our secondary objectives are to synthesize the available evidence on the relationship between the cumulative dose of naloxone administered and (1) the proportion of patients experiencing effective reversal of opioid toxicity and (2) the proportion of patients experiencing serious adverse events in cases of non-medical opioid use for undifferentiated opioid overdoses and in cases of suspected ultra-potent opioid toxicity. We will answer these questions for naloxone administration in both out-of-hospital and in-hospital settings.

## Methods/design

We have followed PRISMA-P guidelines for the reporting of this protocol (Additional file 3) [64]. As we aim to address the limitations of recent systematic reviews that excluded data on ultra-potent opioids, we have tailored our methods to capture available data on these agents. We will document protocol changes as amendments adhering to PRISMA-P guidelines.

### Eligibility criteria

#### Population

The population of interest are people who suffered from suspected or confirmed opioid toxicity (manifested by respiratory depression, depressed level of consciousness, and/or cardio-respiratory arrest) induced by suspected non-medical use of opioids and ultra-potent opioids in particular. We will not base inclusion on the specific type of opioid used and will include mixed intoxications, as information about specific agents ingested is usually unavailable to bystanders and medical personnel at the time of treatment. We will include data from children over the age of 12, as we suspect that teenagers who use opioids are close to adults in height and weight and would receive the same recommended treatment strategy as adults.

#### Intervention

 The intervention of interest is the administration of an empiric naloxone dose (and possible additional naloxone doses) for the treatment of suspected or confirmed opioid toxicity from non-medical opioid use. We will include studies reporting naloxone use for any confirmed or suspected opioid ingestion by any route, which includes known ultra-potent opioids, opioids originally obtained by prescription, tampered with, and/or illicitly manufactured. We will examine naloxone use by all routes of administration and will include reports of naloxone administration by both lay and healthcare personnel in both out-of-hospital and in-hospital settings.

#### Comparator

None.

#### Outcomes

The three main outcomes after naloxone administration are as follows: (1) clinical reversal of opioid toxicity, defined by but not limited to the return of spontaneous breathing, an increase in respiratory rate, return of consciousness, or discharge alive from medical care; (2) occurrence of serious adverse effects, including but not limited to acute opioid withdrawal, pulmonary edema, and seizures; and (3) cumulative dose of naloxone administered.

### Study design

We will include randomized and non-randomized controlled trials, non-comparator trials, prospective and historical cohort studies, cross-sectional studies, and case-control studies. Given the recently changing epidemiology of opioid use and the high likelihood that the treatment of ultra-potent opioid overdoses may not yet have been described in formal studies, we will also include case series, case reports, and reports from poison centers [65]. We will retain editorials, commentaries, letters, and reviews identified by our search: we will not formally include these in our systematic review, however, they will assist us in identifying additional relevant eligible studies and important relevant gray literature sources.

### Search strategy

**Fig. 1:**
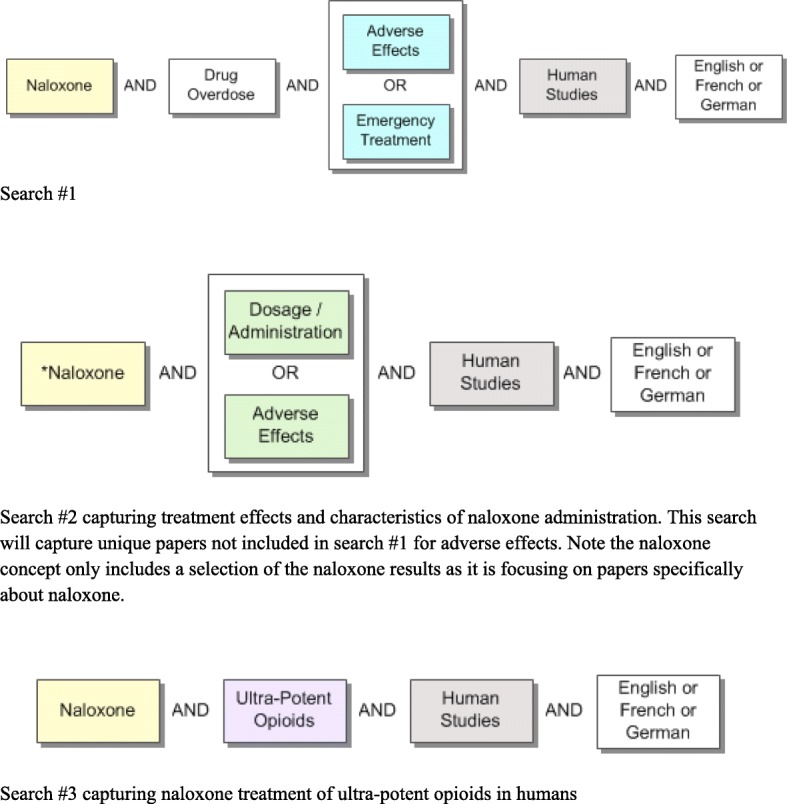
Search concepts for MEDLINE (Ovid)

**Fig. 2:**
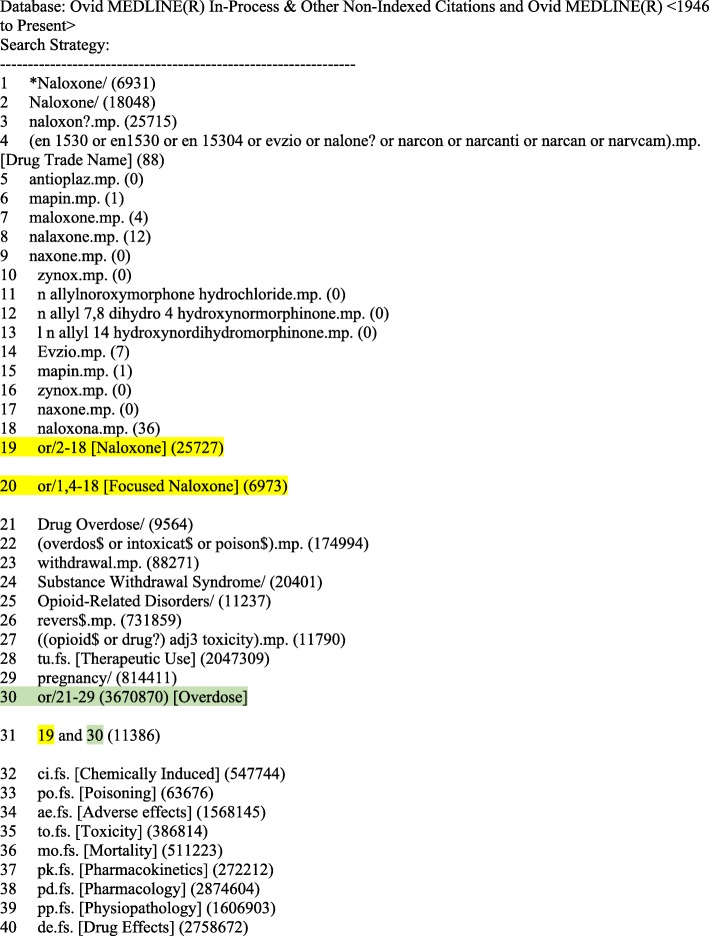
Draft MEDLINE (Ovid) search strategy

We will develop a systematic search strategy with a professional librarian (MDW) with two parallel aims: first, to capture the dosing, effectiveness, and adverse effects of naloxone in all opioid overdoses, and, second, to capture all available evidence specifically pertaining to ultra-potent opioids. Our three preliminary searches will be combined and include the following concepts: (1) *naloxone* AND *drug overdose* AND (*adverse effects* OR *emergency treatment)*; (2) *naloxone* AND (*adverse effects* OR *dosage/administration*); and (3) *naloxone* AND *ultra-potent opioids* (see Additional file 1 Search Concepts). We will develop sensitive searches using applicable subject headings and keywords to capture as much of the relevant literature as possible. We have reviewed papers on naloxone for relevant subject headings and keywords to include in our search. We will also review the scope notes for all subject headings to ensure inclusion of all pertinent terms and prior indexing terms and will identify and include appropriate synonyms for all of the ultra-potent opioids identified so far (Table 1). A draft MEDLINE (Ovid) search is included (see Additional file 2, Draft MEDLINE (Ovid) Search Strategy) (Figs. 1 and 2). We will use our MEDLINE search as a starting point for adaptation to other databases and will iteratively refine and update our searches. We will not restrict our searches by language; we will examine abstracts in all languages but will only include studies published in English, French, or German for full-text review. We will include studies on naloxone and opioids published after 1972, as this is when naloxone and fentanyl entered the legal market.

**Table 1:** Opioids involved in overdose deaths in Canada [19]

Opioids involved in overdose deaths in Canada	
Fentanyl-related opioids	• Fentanyl
	• Carfentanil
	• Norfenanyl
	• Acetylfentanyl
	• Butyryfentanyl
	• Furanyl-fentanyl
	• 3-methylfentanyl
	• Despropinyl-fentanyl
Non-fentanyl related opioids	• Heroin
	• Codeine
	• U-47700
	• Tramadol
	• Morphine
	• Tapentadol
	• Oxycodone
	• Loperamide
	• Meperidine
	• Methadone
	• Hydrocodone
	• Normeperidine
	• Dihydrocodeine
	• Hydromorphone
	• Monoacetylmorphine
	• Buprenorphine metabolites

### Information sources

We will search the following electronic reference databases: MEDLINE, Embase, Cochrane Central Register of Controlled Trials (CENTRAL), Database of Abstracts of Reviews of Effects (DARE) all available through Ovid, Cochrane Drugs and Alcohol Group (CDAG) Specialized Register, CINAHL - Cumulative Index to Nursing and Allied Health Literature through EBSCO, and the Science Citation Index (Web of Science Core Collection) from Clarivate Analytics. We also will review reference lists and trial registries for unpublished trials, including the ISRCTN Registry, ClinicalTrials.gov, EU Clinical Trials Register and South African National Clinical Trials Register, Open Trials, and the Quebec Pain Registry. We will search for studies meeting our inclusion criteria using the Web of Science Core Collection and ScienceDirect (Elsevier).

We will perform an electronic gray literature search using the search engine Google by combining relevant search terms from our bibliographic database search. In addition, we will search the websites of professional organizations, harm reduction initiatives, and of international, national, and provincial guidelines for the treatment of opioid overdose (Table 2). We will search conference proceedings through database searches in Embase (Ovid) and the Web of Science. Additionally, we will specifically search the conference proceedings of the International Society for the Study of Drug Policy (ISSDP), National Harm Reduction Conference, Issues of Substance (IOS), and the International Harm Reduction Conference. We will also hand search the table of contents of relevant journals, for example, the Canadian Journal of Emergency Medicine and International Journal of Drug Policy.

**Table 2:** List of relevant professional organizations

List of professional organizations	
The Canadian Association of Poison Control Centers	
The American Association of Poison Control Centers	
The American College of Medical Toxicology	
The American Academy of Clinical Toxicology	
The European Association of Poison Centers and Clinical Toxicology	
The Asian Pacific Association of Medical Toxicology	
European Monitoring Centre for Drugs and Drug Addiction	
National Institute of Drug Abuse	
National Drug and Alcohol Research Centre	
Advisory Council on the Misuse of Drugs	

### Study records

#### Data management

We will create a search report of all searches and their sources and will capture all identified titles in RefWorks. We will use unique folders for each step of the search process within a common team RefWorks account. For our gray literature searches, we will track the search terms and the date of the searches performed.

#### Selection of studies

Two reviewers will independently review the titles and abstracts of identified references for eligibility based on the inclusion and exclusion criteria. Both reviewers will pilot test the inclusion and exclusion criteria on a convenience sample of the first 15 titles in our search results, to ensure we have adequately described and are able to consistently apply our study selection criteria. All potentially relevant titles identified by both reviewers will be moved forward for full-text review. Any disagreements relating to the inclusion or exclusion of full-text articles between the two reviewers will be resolved through discussion or, if required, a third reviewer. We will document inclusion and exclusion decisions on a study selection form. We will record and report the reason for excluding records.

One reviewer will review the first 100 results of our Google search result pages for relevant reports and literature and will move the full texts of any relevant documents, reports, or websites identified in our Google search forward for full-text review. Two reviewers will then independently review all potentially relevant full texts as per our eligibility criteria.

### Data collection process

Two reviewers will independently extract data from each included study using a study/data collection form. We will resolve any disagreements through discussion until achieving consensus or, if required, a third reviewer. Both reviewers will pilot test data collection on the first 15 included studies to ensure that the form does not require revision and that data extraction is consistent.

#### Data items

We will extract data on relevant information about the type of opioid ingested (if known), study date and location, the study participants, the geographic location and timing of patient enrolment (which we will use to estimate prevalence of ultra-potent opioids), the intervention (both experimental and control for experimental studies), including information about initial and subsequent doses, route, frequency, and sequence of naloxone administration, as well as the person and setting of its administration, and patient outcomes. We will collect information on study design, participants, setting, data quality, limitations, and funding sources. We will contact study authors for missing information or clarifications required for data synthesis. We will attempt to contact authors by email at least twice with emails sent three weeks apart.

#### Risk of bias in individual studies

Two reviewers will independently appraise each included study for potential sources of bias. We will appraise observational studies using an adaptation of the Downs and Black risk of bias assessment tool modified for observational studies. We will assess randomized controlled trials with the Cochrane Risk of Bias Tool to assess for selection, performance, attrition and reporting bias, and possible conflicts of interest. The two reviewers will resolve all disagreements by discussing until reaching consensus. If they cannot reach consensus, a third reviewer will adjudicate the study.

### Data synthesis

Based on recent studies and systematic reviews in this field, we anticipate that many studies will report outcomes as proportions of patients who experience clinical reversal of opioid toxicity by dose level [63, 66]. In our meta-analysis (if performed), we will deconstruct any comparative studies into its one-arm components and use the aggregate results by arm. We will plot log (proportion) versus dose to graphically summarize the dose-response relationship for naloxone administration by similar route of administration; the symbol size will be proportional to the precision of the estimate. We will use a random effect, one-stage meta-regression to estimate the dose-response relationship. If we find a sufficient number of distinct doses, we will use a flexible method (splines) to model dose; with only a few distinct doses, we will adopt an unstructured dose trend treating dose as a categorical variable. We will adopt a similar strategy for the proportion of patients experiencing a serious adverse event. A sensitivity analysis will be conducted to exclude studies that are categorized as high risk of bias.

We will assess heterogeneity using the *I*^2^ statistic. Regardless of the magnitude of *I*^2^, we will explore clinical heterogeneity by adding covariates to the dose-response meta-regression. As we anticipate a limited number of studies, we will incorporate only one variable at a time. Variables to be investigated include the following: the type of opioid used (non-ultra-potent versus ultra-potent, mixed versus single agent), time, geographic location (with suspected high and low prevalence of ultra-potent opioids), the setting of naloxone use (e.g., hospital, pre-hospital, bystander), its route of administration (e.g., intravenous, intranasal, intramuscular), administering provider (layperson versus medical personnel), and frequency or schedule of naloxone administration. If we find insufficient studies for meta-analysis, we will synthesize the information qualitatively.

We will interpret our findings in light of the suspected prevalence of ultra-potent opioids based on year and geographic location of the studies examined. We will define effectiveness as the clinical reversal of opioid toxicity after naloxone administration as indicated, for example, by increased respiratory effort or rate, improved level of consciousness, return of spontaneous circulation, and/or discharge alive from medical care. We will define serious adverse events by the occurrence of adverse clinical responses to naloxone including but not limited to acute opioid withdrawal syndrome, pulmonary edema, and seizures. We will define the total dose used as the sum of all naloxone doses administered to a patient. We will perform subgroup analyses assessing the primary and secondary outcomes listed above within studies based on type of opioid ingested, by varying prevalence of ultra-potent opioids based on time and geographic location, by setting of naloxone administration (bystander, pre-hospital, hospital), by route of naloxone administration, and by frequency or schedule of naloxone administration.

### Confidence in cumulative evidence

We will present the results of our meta-analysis (if performed) using a GRADE summary of findings table. This table will present the summarized naloxone dose-response relationship alongside a score for the quality of the evidence used to generate that value. We will assign the quality of evidence scores based on the number and quality of the component studies and the consistency and generalizability within them.

### Knowledge translation

We will disseminate our results through traditional academic mechanisms, including abstracts to national and international meetings and open-access peer-reviewed publications. We will produce briefing notes from our findings to inform health care professionals, policy makers, and patient safety organizations. We will immediately disseminate our results to all emergency physicians in BC, where the epicenter of the current epidemic is, through the province-wide Emergency Medicine Network of which several of our authors are members. Furthermore, our results will be shared widely with emergency physicians in Canada through research bulletins published by the Canadian Association of Emergency Physicians. In addition, our results will inform the training, naloxone dosing, and administration schedule in BC Take Home Naloxone Kits being widely disseminated to people who use opioids, family and friends of people who use opioids, public bystanders, and staff involved in responding to opioid overdoses outside of acute care settings. Our results will also inform program training resources available to the public through the BC Centre for Disease Control’s “Toward the Heart” program (available at towardtheheart.com). These program training resources include manuals, brochures, posters, videos, online applications, and modules which train members of the public on how to effectively respond to opioid overdoses. Furthermore, results and knowledge translation resources will be presented and made available to the BC Drug Overdose & Alert Partnership (DOAP), a multi-sectoral committee that includes emergency health services and regional health authorities. Tailored information materials will be disseminated to Individuals in observed drug use settings, such as overdose prevention services and supervised consumption services sites.

## Discussion

The government of Canada’s Joint Statement of Action to address the current opioid crisis identifies naloxone administration as a key pillar [67]. Improving naloxone access by removing the need for a prescription and making it available free of charge, as have been done in Canada and BC, is an important example from which all of North America should learn [68]. However, in order for naloxone administration to be safe and effective when used to treat ultra-potent opioid overdoses, we must determine the appropriate dose and route of its administration. Our review is specifically designed to address the limitations of prior reviews and to include the most up-to-date available data to provide an answer to these urgent questions. In contrast to standard systematic reviews, we will deliberately seek non-traditional sources of data from case reports, case series, and poison control center reports. This will enable us to obtain a nuanced understanding of naloxone effectiveness based on broad inclusion of available evidence. Our results will be used to inform practice to reduce the toxicity that continues to claim over 100 lives in BC alone every month [14].

Potential limitations of our review will be the inclusion of only English, French, and German publications and the anticipated heterogeneity of studies that may limit our ability to perform a traditional meta-analysis. However, our broad inclusion of multiple information sources including the most recent studies and reports on naloxone administration will allow us to synthesize all of the currently available evidence on this topic to inform naloxone dosing in the evolving and unabated opioid crisis.

## Additional files

Additional file 1: Search Concepts for MEDLINE (Ovid). (DOCX 26 kb)

Additional file 2: Draft MEDLINE (Ovid) Search Strategy. (DOCX 17 kb)

Additional file 3: PRISMA-P 2015 Checklist. (DOCX 15 kb)

## Data Availability

Not applicable.

## Abbreviations

BC: British Columbia
CENTRAL: Cochrane Central Register of Controlled
CDAG: Cochrane Drugs and Alcohol Group
DARE: Database of Abstracts of Reviews of Effects
DOAP: BC Drug Overdose & Alert Partnership
ISSDP: International Society for the Study of Drug Policy
IOS: Issues of Substance

## References

[1] Pathan H, Williams J (2012). Basic opioid pharmacology: an update. Br J Pain.

[2] Trescot AM, Datta S, Lee M, Hansen H (2008). Opioid pharmacology. Pain Physician.

[3] Fields HL (2011). The doctor’s dilemma: opiate analgesics and chronic pain. Neuron.

[4] World Health Organization. WHO's cancer pain ladder for adults. 2019. Available at: https://www.who.int/cancer/palliative/painladder/en/.

[5] van Dorp ELA, Yassen A, Dahan A (2007). Naloxone treatment in opioid addiction: the risks and benefits. Expert Opin Drug Saf.

[6] Wise RA, Bozarth MA (1985). Brain mechanisms of drug reward and euphoria. Psychiatr Med.

[7] Volkow ND, McLellan AT (2016). Opioid abuse in chronic pain - misconceptions and mitigation strategies. N Engl J Med.

[8] Fischer B, Jones W, Rehm J (2014). Trends and changes in prescription opioid analgesic dispensing in Canada 2005-2012: an update with a focus on recent interventions. BMC Health Serv Res.

[9] Gomes T, Mamdani MM, Dhalla IA, Cornish S, Paterson JM, Juurlink DN (2014). The burden of premature opioid-related mortality. Addiction.

[10] Dart RC, Severtson SG, Bucher-Bartelson B (2015). Trends in opioid analgesic abuse and mortality in the United States. N Engl J Med.

[11] Paulozzi LJ, Budnitz DS, Xi YL (2006). Increasing deaths from opioid analgesics in the United States. Pharmacoepidemiol Drug Saf.

[12] Smolina K, Gladstone E, Morgan SG (2016). Determinants of trends in prescription opioid use in British Columbia, Canada, 2005–2013. Pharmacoepidemiol Drug Saf.

[13] Morin KA, Eibl JK, Franklyn AM, Marsh DC (2017). The opioid crisis: past, present and future policy climate in Ontario, Canada. Subst Abuse Treat Prev Policy.

[14] Fentanyl-Detected Illicit Drug Overdose Deaths January 1, 2012 to September 30, 2017. In: Edited by Service BCs. Burnaby: Ministry of Public Safety and Solicitor General; 2017.

[15] Alberta Health. Opioids and Substances of Misuse: Alberta Report, 2017 Q1. Government of Alberta: Alberta; 2017.

[16] Boyer EW (2012). Management of opioid analgesic overdose. N Engl J Med.

[17] Worley J (2017). A primer on heroin and fentanyl. J Psychosoc Nurs Ment Health Serv.

[18] Green TC, Gilbert M (2016). Counterfeit medications and fentanyl. JAMA Intern Med.

[19] Special Advisory Committee on the Epidemic of Opioid Overdoses. National report: Apparent opioid-related deaths in Canada (January 2016 to March 2017). Web Based Report. Ottawa: Public Health Agency of Canada; 2017. https://health-infobase.canada.ca/datalab/national-surveillance-opioid-mortality.html.

[20] Service BC (2018). Fentanyl-detected illicit drug overdose deaths January 1, 2012 to March 31, 2018.

[21] Government of Canada. Drug Analysis Service: Summary report of samples analysed. Ottawa: Government of Canada; 2018. Available from: https://www.canada.ca/en/health-canada/services/health-concerns/c.ontrolled-substances-precursor-chemicals/drug-analysis-service/drug-analysis-service-summary-report-samples-analysed.html.

[22] Ngai SH, Berkowitz BA, Yang JC, Hempstead J, Spector S (1976). Pharmacokinetics of naloxone in rats and in man: basis for its potency and short duration of action. Anesthesiology.

[23] Sporer KA, Dorn E (2001). Heroin-related noncardiogenic pulmonary edema - a case series. Chest.

[24] Reed CR, Glauser FL (1991). Drug-induced noncardiogenic pulmonary-edema. Chest.

[25] Duberstein JL, Kaufman DM (1971). Clinical study of an epidemic of heroin intoxication and heroin-induced pulmonary edema. Am J Med.

[26] Nath SS, Tripathi M, Pandey C, Rao B (2009). Naloxone-induced pulmonary edema: a potential cause of postoperative morbidity in laparoscopic donor nephrectomy. Indian J Med Sci.

[27] Johnson C, Mayer P, Grosz D (1995). Pulmonary-edema following naloxone administration in a healthy orthopedic patient. J Clin Anesth.

[28] Partridge BL, Ward CF (1986). Pulmonary-edema following low-dose naloxone administration. Anesthesiology.

[29] Flacke JW, Flacke WE, Williams GD (1977). Acute pulmonary-edema following naloxone reversal of high-dose morphine anesthesia. Anesthesiology.

[30] Prough DS, Roy R, Bumgarner J, Shannon G (1984). Acute pulmonary-edema in healthy teenagers following conservative doses of intravenous naloxone. Anesthesiology.

[31] Taff RH (1983). Pulmonary-edema following naloxone administration in a patient without heart-disease. Anesthesiology.

[32] Schwartz JA, Koenigsberg MD (1987). Naloxone-induced pulmonary-edema. Ann Emerg Med.

[33] Michaelis LL, Hickey PR, Clark TA, Dixon WM (1974). Ventricular irritability associated with the use of naloxone hydrochloride. Two case reports and laboratory assessment of the effect of the drug on cardiac excitability. Ann Thorac Surg.

[34] Kim HK, Nelson LS (2015). Reducing the harm of opioid overdose with the safe use of naloxone: a pharmacologic review. Expert Opin Drug Saf.

[35] Cuss FM, Colaco CB, Baron JH (1984). Cardiac-arrest after reversal of effects of opiates with naloxone. Br Med J.

[36] Andree RA (1980). Sudden-death following naloxone administration. Anesth Analg.

[37] Buajordet I, Naess A-C, Jacobsen D, Brors O (2004). Adverse events after naloxone treatment of episodes of suspected acute opioid overdose. Eur J Emerg Med.

[38] Sivilotti MLA (2016). Flumazenil, naloxone and the “coma cocktail”. Br J Clin Pharmacol.

[39] Popper C, Kelen GD, Cunningham G (1989). Naloxone hazard in drug-abuser. Lancet.

[40] Gaddis GM, Watson WA (1992). Naloxone-associated patient violence - an overlooked toxicity. Ann Pharmacother.

[41] Cook S, Moeschler O, Michaud K, Yersin B (1998). Acute opiate overdose: characteristics of 190 consecutive cases. Addiction.

[42] Caudarella A, Dong HR, Milloy MJ, Kerr T, Wood E, Hayashi K (2016). Non-fatal overdose as a risk factor for subsequent fatal overdose among people who inject drugs. Drug Alcohol Depend.

[43] Seidler D, Stuhlinger GH, Fischer G, Woisetschlaeger C, Berzlanovich A, Schmid R, Hirschl MM, Laggner AN (1996). After antagonization of acute opiate overdose: a survey at hospitals in Vienna. Addiction.

[44] Klassen D (2016). Overdose recognition and response in the BC Take Home Naloxone Program.

[45] Lavonas Eric J., Drennan Ian R., Gabrielli Andrea, Heffner Alan C., Hoyte Christopher O., Orkin Aaron M., Sawyer Kelly N., Donnino Michael W. (2015). Part 10: Special Circumstances of Resuscitation. Circulation.

[46] Connors NJ, Nelson LS (2016). The evolution of recommended naloxone dosing for opioid overdose by medical specialty. J Med Toxicol : official journal of the American College of Medical Toxicology.

[47] Evans JM, Hogg MIJ, Lunn JN, Rosen M (1974). Degree and duration of reversal by naloxone of effects of morphine in conscious subjects. Br Med J.

[48] Schumann H, Erickson T, Thompson TM, Zautcke JL, Denton JS (2008). Fentanyl epidemic in Chicago, Illinois and surrounding Cook County. Clin Toxicol.

[49] Christenson J, Etherington J, Grafstein E, Innes G, Pennington S, Wanger K, Fernandes C, Spinelli JJ, Gao M (2000). Early discharge of patients with presumed opioid overdose: development of a clinical prediction rule. Acad Emerg Med.

[50] Sutter ME, Gerona RR, Davis MT, Roche BM, Colby DK, Chenoweth JA, Adams AJ, Owen KP, Ford JB, Black HB (2017). Fatal fentanyl: one pill can kill. Acad Emerg Med.

[51] Kerr D, Kelly A-M, Dietze P, Jolley D, Barger B (2009). Randomized controlled trial comparing the effectiveness and safety of intranasal and intramuscular naloxone for the treatment of suspected heroin overdose. Addiction.

[52] Kelly AM, Kerr D, Dietze P (2005). Randomised trial of intranasal versus intramuscular naloxone in prehospital treatment for suspected opioid overdose - reply. Med J Aust.

[53] Robertson TM, Hendey GW, Stroh G, Shalit M (2009). Intranasal naloxone is a viable alternative to intravenous naloxone for prehospital narcotic overdose. Prehosp Emerg Care.

[54] CADTH (2017). Intranasal and intramuscular naloxone for opioid overdose in the pre-hospital setting: a review of comparative clinical and cost-effectiveness, and guidelines. CADTH rapid response report: summary with critical apprasial.

[55] Clarke SFJ, Dargan PI, Jones AL (2005). Naloxone in opioid poisoning: walking the tightrope. Emerg Med J.

[56] Stolbach A, Traub SJ, J G (2016). Acute opioid intoxication in adults.

[57] Kuhlman JJJ, McCaulley R, Valouch TJ, Behonick GS (2003). Fentanyl use, misuse, and abuse: a summary of 23 postmortem cases. J Anal Toxicol.

[58] Jones TS, Krzywicki L, Maginnis J, Jones NL, Weiskopf R, Reid M, Schmidt C, Fiedler J, Topolski JM, Graham M (2008). Nonpharmaceutical fentanyl-related deaths - multiple states, April 2005-March 2007 (Reprinted MMWR, vol 57, pg 793-796, 2008). Jama-J Am Med Assoc.

[59] Martin TL, Woodall KL, McLellan BA (2006). Fentanyl-related deaths in Ontario, Canada: toxicological findings and circumstances, of death in 112 cases (2002-2004). J Anal Toxicol.

[60] Willman MW, Liss DB, Schwarz ES, Mullins ME (2017). Do heroin overdose patients require observation after receiving naloxone?. Clin Toxicol.

[61] Strang J, McDonald R, Tas B, Day E (2016). Clinical provision of improvised nasal naloxone without experimental testing and without regulatory approval: imaginative shortcut or dangerous bypass of essential safety procedures?. Addiction.

[62] Mueller SR, Walley AY, Calcaterra SL, Glanz JM, Binswanger IA (2015). A review of opioid overdose prevention and naloxone prescribing: implications for translating community programming into clinical practice. Subst Abus.

[63] Chou R, Korthuis P, McCarty D (2017). Management of suspected opioid overdose with naloxone in out-of-hospital settings: a systematic review. Ann Intern Med.

[64] Moher D, Liberati A, Tetzlaff J, Altman DG (2009). The PG: Preferred Reporting Items for Systematic Reviews and Meta-Analyses: The PRISMA Statement. PLoS Med.

[65] Stroup DF, Berlin JA, Morton SC (2000). Meta-analysis of observational studies in epidemiology: a proposal for reporting. JAMA.

[66] Kim HK, Nelson LS (2016). Reversal of opioid-induced ventilatory depression using low-dose naloxone (0.04 mg): a case series. J Med Toxicol.

[67] Canada Go. Joint statement of action to address the opioid crisis: Government of Canada; 2016.

[68] Government of Canada. Joint Statement of Action to Address the Opioid Crisis. Ottawa: Government of Canada; 2016.

