# Supplementary material for: Naloxone interventions in opioid overdoses: a systematic review protocol
Source: Syst Rev. 2019 Jun 11;8:138. doi: 10.1186/s13643-019-1048-y (PMC6560883; doi:10.1186/s13643-019-1048-y)
Supplement: Supplementary file 1 — Search Concepts for MEDLINE (Ovid). (DOCX 26 kb) [file 13643_2019_1048_MOESM1_ESM.docx]

**Additional File 1.** Search Concepts for MEDLINE (Ovid)


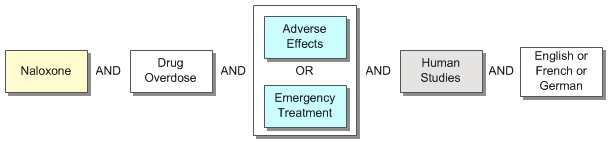


Search #1


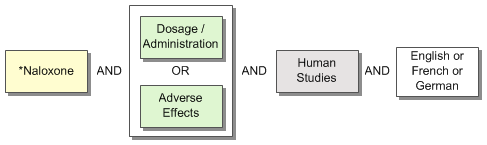


Search #2 capturing treatment effects and characteristics of naloxone administration. This search will capture unique papers not included in search #1 for adverse effects. Note the naloxone concept only includes a selection of the naloxone results as it is focusing on papers specifically about naloxone.


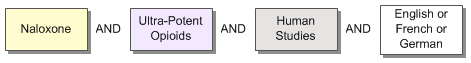


Search #3 capturing naloxone treatment of ultra-potent opioids in humans
