# Supplementary material for: Naloxone interventions in opioid overdoses: a systematic review protocol
Source: Syst Rev. 2019 Jun 11;8:138. doi: 10.1186/s13643-019-1048-y (PMC6560883; doi:10.1186/s13643-019-1048-y)
Supplement: Supplementary file 2 — Draft MEDLINE (Ovid) Search Strategy. (DOCX 17 kb) [file 13643_2019_1048_MOESM2_ESM.docx]

**Additional File 2.** Draft MEDLINE (Ovid) Search Strategy

Database: Ovid MEDLINE(R) In-Process & Other Non-Indexed Citations and Ovid MEDLINE(R) <1946 to Present>

Search Strategy:

----------------------------------------------------------------

1 *Naloxone/ (6931)

2 Naloxone/ (18048)

3 naloxon?.mp. (25715)

4 (en 1530 or en1530 or en 15304 or evzio or nalone? or narcon or narcanti or narcan or narvcam).mp. [Drug Trade Name] (88)

5 antioplaz.mp. (0)

6 mapin.mp. (1)

7 maloxone.mp. (4)

8 nalaxone.mp. (12)

9 naxone.mp. (0)

10 zynox.mp. (0)

11 n allylnoroxymorphone hydrochloride.mp. (0)

12 n allyl 7,8 dihydro 4 hydroxynormorphinone.mp. (0)

13 l n allyl 14 hydroxynordihydromorphinone.mp. (0)

14 Evzio.mp. (7)

15 mapin.mp. (1)

16 zynox.mp. (0)

17 naxone.mp. (0)

18 naloxona.mp. (36)

19 or/2-18 [Naloxone] (25727)

20 or/1,4-18 [Focused Naloxone] (6973)

21 Drug Overdose/ (9564)

22 (overdos$ or intoxicat$ or poison$).mp. (174994)

23 withdrawal.mp. (88271)

24 Substance Withdrawal Syndrome/ (20401)

25 Opioid-Related Disorders/ (11237)

26 revers$.mp. (731859)

27 ((opioid$ or drug?) adj3 toxicity).mp. (11790)

28 tu.fs. [Therapeutic Use] (2047309)

29 pregnancy/ (814411)

30 or/21-29 (3670870) [Overdose]

31 19 and 30 (11386)

32 ci.fs. [Chemically Induced] (547744)

33 po.fs. [Poisoning] (63676)

34 ae.fs. [Adverse effects] (1568145)

35 to.fs. [Toxicity] (386814)

36 mo.fs. [Mortality] (511223)

37 pk.fs. [Pharmacokinetics] (272212)

38 pd.fs. [Pharmacology] (2874604)

39 pp.fs. [Physiopathology] (1606903)

40 de.fs. [Drug Effects] (2758672)

41 co.fs. [Complications] (1825065)

42 "drug-related side effects and adverse reactions"/ or drug hypersensitivity/ or "chemical and drug induced liver injury"/ (75059)

43 interaction?.mp. (1222770)

44 apnea/ (6874)

45 coma/ (11987)

46 Respiratory Insufficiency/ (29817)

47 Myocardial Contraction/ (51309)

48 Death, Sudden/ (11876)

49 SEIZURES/ (49177)

50 Heart Arrest/ (26662)

51 arrhythmias, cardiac/ (58055)

52 pulmonary edema/ (16570)

53 hypertension/ (219620)

54 respiratory depression.mp. (5573)

55 poisoning/ (21667)

56 Suicide, Attempted/ (17883)

57 Obstetric Labor Complications/ (16110)

58 RESPIRATION/ (75698)

59 death.mp. (685480)

60 side effect?.mp. (246257)

61 (adverse adj8 (event? or effect? or reaction? or outcome?)).mp. (381337)

62 complication?.mp. (1233337)

63 exp Drug Interactions/ (152472)

64 violence/ (28269)

65 resuscitation/ (24607)

66 treatment failure/ (31750)

67 (treatment adj3 failure).mp. (58981)

68 or/32-67 (10262524) [Adverse Effects]

69 19 and 30 and 68 (10033) [Naloxone & Overdose & AEs]

70 Emergency Treatment/ (9892)

71 Emergency Medical Services/ (38441)

72 emergencies/ (38073)

73 emergenc$.mp. (366527)

74 Emergency medicine/ (12053)

75 hospitalization/ (92286)

76 first aid/ (7479)

77 emergency service, hospital/ or trauma centers/ (65516)

78 Patient Care Team/ (60151)

79 emergency responders/ or emergency medical technicians/ or firefighters/ or police/ (10816)

80 Ambulances/ (5631)

81 ambulance?.mp. (13311)

82 first responder?.mp. (1622)

83 (layperson? or lay person? or lay responder?).mp. (1925)

84 Community Health Services/ (29721)

85 Community mental health services/ (17779)

86 Support provider?.mp. (364)

87 bystander?.mp. (8949)

88 self administration.mp. (14031)

89 self medication/ (4432)

90 Transportation of Patients/ (8791)

91 Pharmacists/ (14119)

92 Physicians/ (80800)

93 Pharmaceutical Services/ (7680)

94 Emergency Medical Services/ (38441)

95 Life support care/ (7475)

96 (prehospital or pre-hospital or outpatient or out-patient).mp. (140103)

97 Treatment Refusal/ (11346)

98 Patient Transfer/ (7255)

99 Risk Assessment/ (225710)

100 Risk Factors/ (717810)

101 risk/ (113939)

102 safety.mp. (451244)

103 recurrence/ (169056)

104 survival rate/ (153555)

105 time factors/ (1117934)

106 Poison Control Centers/ (2635)

107 Case management/ or disease management/ or risk management/ or safety management/ (73442)

108 management.mp. (1068797)

109 Harm Reduction/ (2379)

110 treatment outcome/ (835459)

111 pc.fs. [Prevention and control] (1174626)

112 Disease Outbreaks/ (73054)

113 outbreak?.mp. (111966)

114 or/70-113 (5410286) [Emergency Medicine]

115 19 and 30 and 114 [Emergency Medicine] (3305)

116 ad.fs. [Administration & Dosage] (1293613)

117 drug administration schedule/ (94475)

118 drug dosage calculations/ (1840)

119 drug delivery systems/ (50707)

120 Infusions, Intraosseous/ (658)

121 drug administration routes/ (5311)

122 administration, inhalation/ (28072)

123 administration, intravenous/ or infusions, intravenous/ or injections, intravenous/ (136060)

124 infusions, parenteral/ (25955)

125 administration, oral/ or administration, buccal/ or administration, sublingual/ (134307)

126 administration, topical/ or administration, cutaneous/ (55399)

127 administration, mucosal/ or administration, intranasal/ (13381)

128 injections, intra-arterial/ (9022)

129 injections, intramuscular/ (29665)

130 injections, intravenous/ (80363)

131 injections, subcutaneous/ or injections, intradermal/ or injections, jet/ (37243)

132 dose response relationship, drug/ (381394)

133 drug therapy, combination/ (155391)

134 (intramuscular or intranasal or intravenous or subcutaneous or intraosseous).mp. (557772)

135 nasal.mp. (115115)

136 take home.mp. (2556)

137 (intramuscular$ or intranasal$ or intravenous$ or subcutaneous$ or intraosseous$).mp. (635294)

138 dos$.mp. (1633618)

139 redos$.mp. (271)

140 Intubation, Intratracheal/ (33079)

141 or/116-140 (3123647) [Dosage]

142 20 and 141 [Naloxone & Dosage – Search 2] (**3901**)

143 ae.fs. [Adverse Effects] (1568145)

144 20 and 143 [Naloxone & AEs – Search 2] (**838**)

145 19 and 30 and (68 or 114) [Naloxone & (AEs or EM)-Search 1] (10530)

146 142 or 144 or 145 [Searches 1 & 2] (12619)

147 animals/ not (humans/ and animals/) (4412165)

148 146 not 147 (**5209**)

149 Naloxone/ (18048)

150 naloxon?.mp. (25715)

151 (en 1530 or en1530 or en 15304 or evzio or nalone? or narcon or narcanti or narcan or narvcam).mp. [Drug Trade Name] (88)

152 antioplaz.mp. (0)

153 mapin.mp. (1)

154 maloxone.mp. (4)

155 nalaxone.mp. (12)

156 naxone.mp. (0)

157 zynox.mp. (0)

158 n allylnoroxymorphone hydrochloride.mp. (0)

159 n allyl 7,8 dihydro 4 hydroxynormorphinone.mp. (0)

160 l n allyl 14 hydroxynordihydromorphinone.mp. (0)

161 Evzio.mp. (7)

162 mapin.mp. (1)

163 zynox.mp. (0)

164 naxone.mp. (0)

165 naloxona.mp. (36)

166 or/149-165 [Naloxone] (25727)

167 fentanyl/ (12655)

168 Fentanyl$.mp. (20404)

169 norco.mp. (10)

170 fentanyl iontophoretic transdermal system.mp. (36)

171 transdermal patch.mp. (1675)

172 (abstral or ap 48 or ap48 or duragesic$ or epufen or fentalis or fentamyl or fentanex or fentanyl or fentanyl or instanyl or lazanda or leptanal or mezolar matrix or onsolis or pecfent or r 4263 or r4263 or rapinyl or recuvyra or subsys or transfenta).mp. [Drug Trade Names] (20411)

173 (phentanyl or tanyl).mp. (118)

174 carfentanil.mp. (270)

175 wildnil.mp. [trade name] (0)

176 R30730.mp. (1)

177 (R 31833 or R31833 or R 33799).mp. (3)

178 Carfentanyl?.mp. (9)

179 Carfentanila.mp. (0)

180 4-methoxycarbonylfentanyl.mp. (0)

181 "1 phenethyl 4 (n phenylpropionamido)isonipecotic acid methyl ester".mp. (0)

182 "4 [(1 oxopropyl)phenylamino] 1 (2 phenylethyl) 4 piperidinecarboxylic acid methyl ester".mp. (0)

183 (Sufentanil or Sufentanilo or Sufentanilum or Sufentanyl).mp. (2658)

184 Zalviso.mp. (2)

185 Sufenta.mp. (5)

186 norfenanyl.mp. (0)

187 Acetylfentanyl.mp. (16)

188 acrylfentanyl.mp. (6)

189 4-fluoro-isobutylfentanyl.mp. (0)

190 alfentanyl.mp. (76)

191 Butyryfentanyl.mp. (0)

192 Furanyl-fentanyl.mp. (13)

193 3-methylfentanyl.mp. (71)

194 Despropinyl-fentanyl.mp. (0)

195 U-4700.mp. (0)

196 (Butyr-fentanyl or butyrylfentanyl).mp. (6)

197 (ultra potent adj3 opioid?).mp. (5)

198 U-49900.mp. (4)

199 (tetrahydrofuranylfentanyl or tetrahydrofuranyl fentanyl).mp. (3)

200 ocfentanil.mp. (6)

201 AH-7921.mp. (21)

202 MT-45.mp. (28)

203 sufentanil.mp. (2622)

204 Remifentanil.mp. (4524)

205 W-18.mp. (119)

206 alpha-methylfentanyl.mp. (15)

207 furanylfentanyl.mp. (11)

208 4-fluorobutyrylfentanyl.mp. (1)

209 4-chloroisobutyrylfentanyl.mp. (1)

210 4-fluoroisobutyrylfentanyl.mp. (1)

211 tetrahydrofuranylfentanyl.mp. (2)

212 cyclopentylfentanyl.mp. (2)

213 Designer Drugs/ (1352)

214 Designer Drug?.mp. (2035)

215 (Novel adj2 (Psychoactive or synthetic) adj4 (Substance? or Opioid?)).mp. (298)

216 or/167-215 [Ultra-Potent Opioids] (29791)

217 166 and 216 [Naloxone & Ultra Potent Opioids - Search 3] (1212)

218 animals/ not (humans/ and animals/) (4412165)

219 217 not 218 (594)

220 146 or 217 (13198)

221 animals/ not (humans/ and animals/) (4412165)

222 220 not 221 (5444)

223 limit 222 to (English or French or German) (**5189**)

224 222 not 223 [Other Languages] (255)

225 comment/ or editorial/ or letter/ or news/ (1783987)

226 223 and 225 (**408**)

227 223 not 226 (**4781**)

Note

All three search results are combined
